# Antimicrobial Activity of Chlorhexidine and Herbal Mouthwash Against the Adherence of Microorganism to Sutures After Periodontal Surgery: A Clinical Microbiological Study

**DOI:** 10.7759/cureus.32907

**Published:** 2022-12-24

**Authors:** Jaishree Garg, Shiva Manjunath RG, Saurabh Sinha, Sanjay Ghambhir, Puru Abbey, Mhao P Jungio

**Affiliations:** 1 Department of Periodontology, Institute of Dental Sciences, Bareilly, IND

**Keywords:** mouthwash, colony forming units, sutures, hioratm, chlorhexidine

## Abstract

*Introduction: *Chemical plaque control agents assist to eliminate plaque microorganisms. Therefore, the purpose of this research was to compare the effectiveness of a herbal mouthwash (HiOra^TM^) and a mouthwash containing 0.2% chlorhexidine in preventing bacteria from adhering to sutures in participants who had had periodontal flap surgery.

*Material and methods: *75 patients with chronic periodontitis were included in the study and divided into three groups. Plain water, herbal, and chlorhexidine mouthwashes were given after periodontal surgery, and sutures were removed on the eighthpostoperative day and sent for microbial analysis. Further plaque index (PI) and gingival index (GI) were recorded at baseline and on the eighthpostoperative day.

*Results: *Different aerobic bacterial species were isolated, namely *Staphylococcus aureus* (S. aureus), coagulase-negative staphylococci (Cons), *Escherechia coli* (E. coli), *Klebsiella bacteroides*, and *Streptococcus mutans* (S. mutans). Significant differences were found among colony-forming units (CFUs) of bacterial species in different groups, and it is found that chlorhexidine mouthwash is quite effective against the adherence of microorganisms to sutures after periodontal surgery as compared to the control group and herbal mouthwash group. Herbal mouthwash has less antimicrobial activity when compared to chlorhexidine, but herbal mouthwash is highly effective against microbial adherence to sutures when compared to the control group (plain water). Significant variations between groups are shown in the PI and GI scores, indicating that chlorhexidine is successful in lowering the PI and GI scores and that herbal rinse is also helpful in reducing these scores, but to a lesser extent than chlorhexidine.

*Conclusion: *According to the findings of this research, sutures are at risk for developing bacterial infections. However, the adhesion of microbes to sutures may be decreased with the adjunctive use of antimicrobial medicines such as chlorhexidine and herbal mouthwashes, which will result in improved wound healing. Herbal mouth rinses are effective in killing germs. However, chlorhexidine is far more effective in this regard.

## Introduction

Effective wound closure is important for the success of any surgical procedure [1]. An incomplete closure leads to the separation of edges, providing a potential pathway for bacterial contamination, leading to infection and scarring [2]. The surgical sutures are still the mainstay of a secure wound. A good suture goes a long way toward aesthetic as well as effective wound closure. The material used is determined by the tissue's requirements. An increasing number of materials and suturing techniques are described in the literature [3]. The sutures used in dentoalveolar surgery represent a risk factor for the healing of surgical wounds because they are prone to the adherence of pathogenic bacteria. The accumulation of microorganisms on sutures may serve as a focus for odontogenic infections. Aerobic and anaerobic bacteria, including fusobacterium, peptostreptococcus, prevotella, porphyromonas, streptococcus, and bacteroides, cause these infections [4].

To reduce the risk of surgical site infections, effective antisepsis, appropriate antimicrobial prophylaxis, and the identification of strategies for decreasing wound contamination must be used [5]. Post-operative care of the sutured wound is very important after surgery. Sutured wounds in the oral cavity are kept clean through frequent rinses with either normal saline, chlorhexidine mouth rinse, hydrogen peroxide diluted with saline, herbal mouthwash, or fresh tap water [6,7]. Mouth rinses are commonly used in conjunction with mechanical hygiene measures to help control supragingival plaque, gingivitis, and post-operative infection [8,9].

Chlorhexidine is a bisbiguanide antiseptic active against gram-positive and gram-negative bacteria, facultative anaerobes and aerobes, molds, yeasts, and viruses [10]. Natural products like herbal medicine can be used to both promote health and keep it from getting worse [11]. It is a comprehensive system that uses various remedies derived from plants and their extracts to maintain good oral health. Natural herbs like triphala, tulsi patra, neem, clove oil, pudhina, ajwain, and many more, used either as whole herbs or in combination, have been scientifically proven to be safe against various oral health problems [12].

Organic and synthetic non-resorbable and resorbable suture materials are currently used in surgery within the mouth. Silk has been a favored suture material in oral and periodontal surgeries because of its ease of handling [13]. The tendency for microbial attachment and accumulation on suture material is one of the most important problems in the healing period. Bacteria and debris that attach to or lodge between the suture materials could delay repair and maintain infection [14]. Thus, in the present study, an attempt has been made to compare the efficacy of chlorhexidine, herbal mouthwash, and plain water against the adherence of microorganisms to the suture material after periodontal surgery.

## Materials and methods

The present study was carried out at the Institute of Dental Sciences, Bareilly, India. The ethical clearance for the study was taken from the institute with reference no. IDS/2020/11.

Inclusion criteria were patients aged 25-60 years of age having chronic periodontitis, cooperative patients, systemic healthy patients, patients requiring periodontal surgery, and patients having a minimum of 20 teeth.

Exclusion criteria had patients who have had antibiotic therapy past six months, pregnancy or lactation, psychiatric disorder, and a history of systemic illness.

Study design

**Figure 1:**
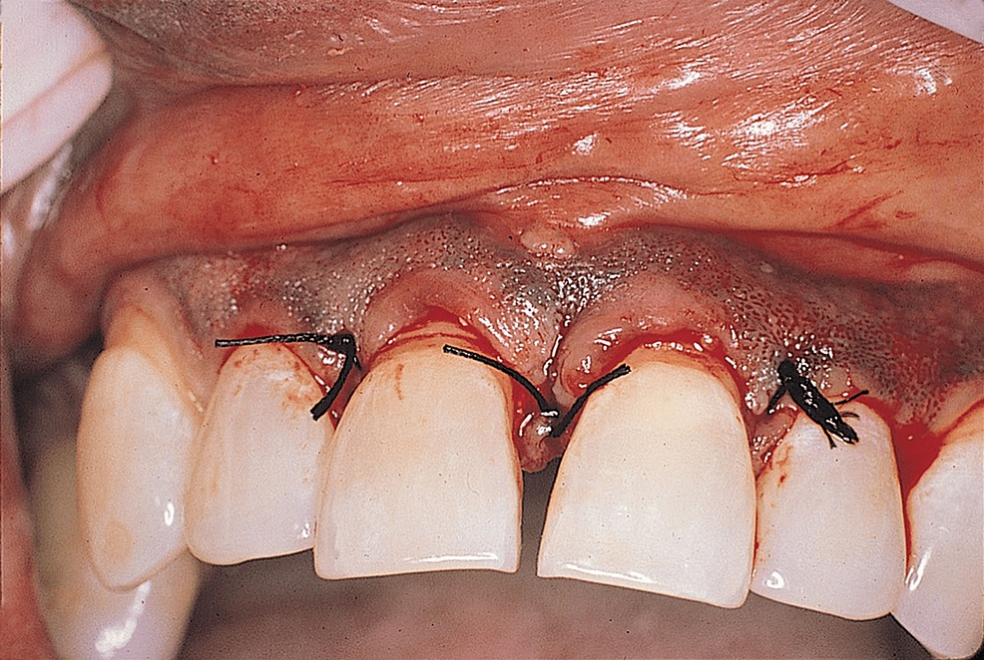
Flap surgery with sutures

The patients were randomly split into three groups of 25 each. At baseline, before flap surgery, and on the eighth day, the periodontal plaque index (PI) and gingival index (GI) were recorded. Patients received scaling, root planning, and oral hygiene instructions as part of Phase 1 treatment. The patients were recalled after four weeks for the periodontal flap surgery. Periodontal flap surgery (Kirkland flap) was performed and sutures were placed with black braided silk sutures (3-0) (Figure 1).

The sutures were removed on the eighth post-operative day under sterile conditions. The suture material was put into a 0.9% sodium chloride (NaCl) saline solution that had been buffered, and then it was taken to the lab for microbiological analysis.

The patients were divided into three groups, with 25 subjects in each group: Group 1 (control) had patients who rinsed with plain water twice daily after periodontal surgery; patients in Group 2 (herbal) were rinsed with 10 ml of herbal (HiOra^TM^) mouthwash twice daily for one minute after surgery; and Group 3 (chlorhexidine 0.2%) had patients who rinsed with 0.2 % chlorhexidine mouth wash 10 ml twice daily for one minute after surgery.

Clinical parameters

The PI and GI were recorded at baseline and on the eighth day postoperatively with the help of an explorer [15].

Microbiological analysis

**Figure 2:**
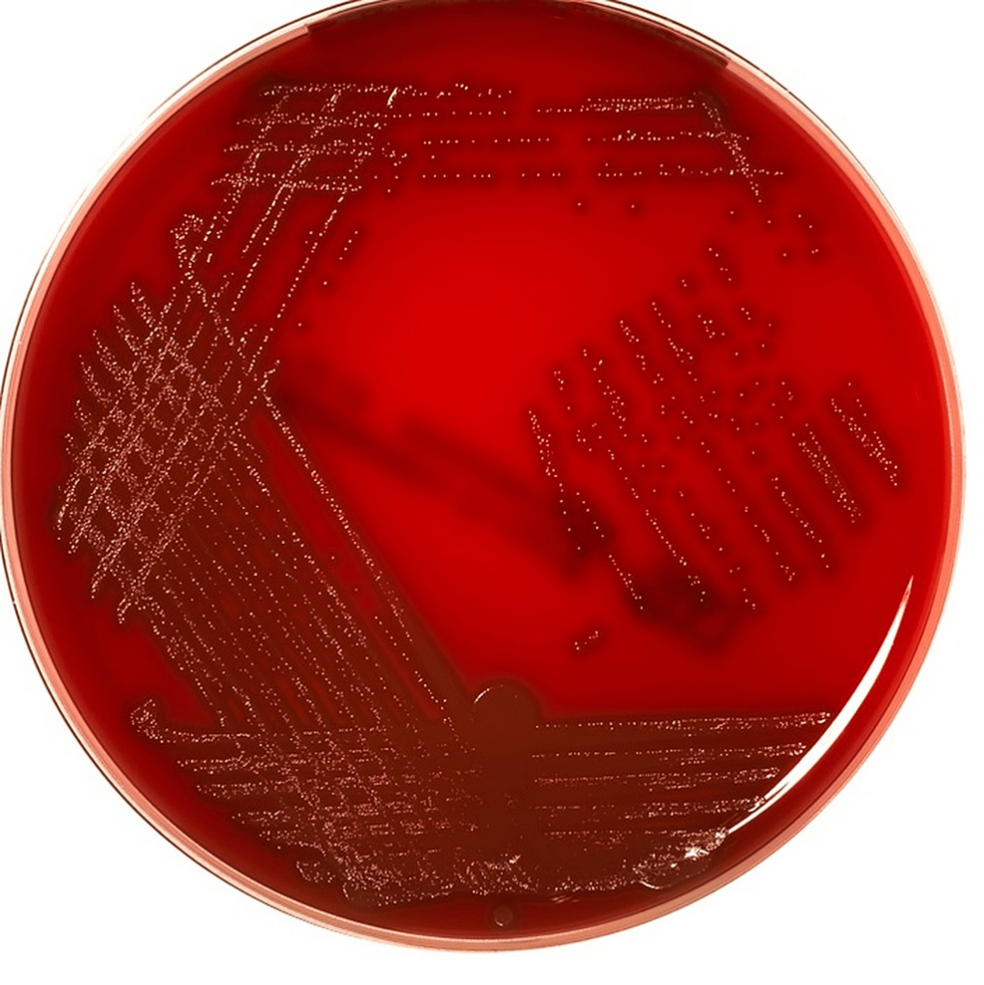
Agar plate with bacterial growth

Inoculation and streaking methods have been done to inoculate the bacteria. The media was autoclaved at 121°C at 15 lbs. Drying of media was done by placing agar plates in an incubator at 37°C for one hour. Following this step, the suture samples are to be put in the medium, one in blood agar, and one in MacConkey agar, in order to determine the gram-positive and gram-negative bacteria using the sticking surface technique. After 24-48 hours in an incubator set at 37°C, the agar plates were removed. A colony counting device can identify the shape of the colony as well as the count of the colony. In order to better view the bacteria under the microscope, gram staining was performed (Figure 2).

Statistical analysis

The statistical analysis was performed using SPSS (statistical package for social sciences) version 24. The data were checked for normal/skewed distribution. The Shapiro-Wilk test indicated that the data are skewed in distribution. The p value was < 0.05, which showed that data is significantly different from a normal distribution. The Kruskal Wallis test was employed for inter-group comparison of colony-forming units (CFUs) of bacteria and scores of GI and PI. Wilcoxon test is employed for within-group comparison of scores of GI and PI before and after the use of an agent.

## Results

There were a total of 75 patients with chronic periodontitis who participated in the trial, and they were randomly assigned to one of three groups: control (plain water), herbal, and chlorhexidine mouthwash. Clinical parameters, including GI and PI, were documented both before and after periodontal surgery. Additional microbiological analysis was performed on Day 8. When PI and GI scores were obtained at baseline and again on Day 8 postoperatively, there were statistically significant differences between the herbal, chlorhexidine, and placebo groups.

In the present study, the staphylococcus species (Table1) is the most dominant species found with a maximum percentage of growth.

**Table 1:** Showing percentage-wise distribution of different bacterial species in patients using plain water, herbal, and chlorhexidine mouth rinses S. aureus: *Staphylococcus aureus*; Cons: Coagulase-negative staphylococci; E. coli: *Escherichia coli*; NPO: Non-pathogenic organism; S. mutans: *Streptococcus mutans*

Groups	S. aureus	Cons	E. coli	NPO	Klebsiella	S. mutans	Total
Plain Water	16%	8%	28%	4%	24%	20%	100%
Herbal	20%	12%	28%	4%	16%	12%	100%
Chlorhexidine	12%	36%	32%	0%	8%	4%	100%

The chlorhexidine group had the minimum microbial adherence to suture with a mean of 2.28, while the herbal had 2.56 and the control group 4.88 (Table2).

**Table 2:** Mean colony count of all bacterial species under study in different groups

Groups	N	Minimum	Maximum	Mean	SD
Plain water	25	4.00	5.00	4.88	0.33
Herbal	25	0.00	4.00	2.56	0.91
Chlorhexidine	25	0.00	4.00	2.28	0.93

The control group (plain water) shows the maximum bacterial colonies with a mean of 4.88, which is reduced to 2.56 in herbal and 2.28 in chlorhexidine on the eighth post-operative day.

In the present study, while chlorhexidine's antibacterial activity was more effective than that of herbal mouthwash (HiOra^TM^), the latter demonstrated a trend toward microbial decrease as well (Table 3).

**Table 3:** Mean CFUs of different bacterial species under study in patients using plain water, herbal, and chlorhexidine mouth rinses CFU: Colony-forming unit; S. aureus: *Staphylococcus aureus*; Cons: Coagulase-negative staphylococci; E. coli: *Escherichia coli*; NPO: Non-pathogenic organism; S. mutans: *Streptococcus mutans*

Group	Bacteria species	CFUs/ml (log_10_)			
		Mean	SD	Minimum	Maximum
Plain water	S. aureus	5.00	0.00	5.00	5.00
	Cons	5.00	0.00	5.00	5.00
	E. coli	5.00	0.00	5.00	5.00
	NPO	5.00		5.00	5.00
	Klebsiella	5.00	0.00	5.00	5.00
	S. mutans	4.40	0.54	4.00	5.00
Herbal	S. aureus	2.33	0.57	2.00	3.00
	Cons	3.00	0.00	3.00	3.00
	E. coli	3.14	0.37	3.00	4.00
	NPO	3.00		3.00	3.00
	Klebsiella	3.50	0.70	3.00	4.00
	S. mutans	3.00	0.00	3.00	3.00
Chlorhexidine	S. aureus	2.20	0.44	2.00	3.00
	Cons	2.66	0.70	2.00	4.00
	E. coli	2.12	0.35	2.00	3.00
	NPO	Nil	nil	Nil	Nil
	Klebsiella	2.50	0.57	2.00	3.00
	S. mutans	2.00	.	2.00	2.00

In the inter-group comparison, statistical significant difference was found between the mean CFU/ml of *Staphylococcus aureus *(S. aureus) (0.012), coagulase-negative staphylococci (Cons) (0.044), *Escherichia coli* (E. coli) (0.000), Klebsiella (0.006), and *Streptococcus mutans *(S. mutans) (0.031) in all control (plain water), herbal, and chlorhexidine groups (Table 4).

**Table 4:** Comparison of bacterial growth (CFU/ml (log10)) in suture materials after the use of plain water, herbal and, chlorhexidine mouth rinses by patients of different groups, respectively *p value is statistically significant. CFU: Colony-forming unit

Type of bacteria	Mean bacterial growth CFU/ml (log_10_)			
	Plain water Mean±SD	Herbal Mean±SD	Chlorhexidine Mean±SD	p. value
S. aureus	5.00±0.00	2.33±0.57	2.20±0.44	0.012*
Cons	5.00±0.00	3.00±0.00	2.66±0.70	0.044*
E. coli	5.00±0.00	3.14±0.37	2.12±0.35	0.000*
NPO	5.00	3.00	nil	0.368
Klebsiella	5.00±0.00	3.50±0.70	2.50±0.57	0.006*
S. mutans	4.40±0.54	3.00±0.00	2.00	0.031*

Inter-group comparison shows significant difference between the mean CFU/ml (log_10_) of S. aureus, Cons, E. coli, Klebsiella, and S. mutans amongst different groups. Post-hoc analysis was performed for further examination.

In the present study, the mean CFUs of S. aureus has been mentioned in Table 5.

**Table 5:** Post-hoc analysis of mean CFU/ml (log10) of S. aureus, Cons, E. coli, Klebsiella, and S. mutans *p value less than 0.05 is considered statistically significant. S. aureus: *Staphylococcus aureus*; CNO: Coagulase-negative staphylococci; E. coli: *Escherichia coli*; S. mutans: *Streptococcus mutans; *CFU: Colony-forming unit

	S. aureus	Cons	E. coli	Klebsiella	S. mutans
Herbal vs chlorhexidine	0.786	0.482	0.002*	0.267	0.500
Chlorhexidine vs plain water	0.016*	0.036*	0.000*	0.010*	0.033*
Plain Water vs herbal	0.047*	0.200	0.001*	0.071	0.036*

Statistical significance is assumed when the p-value is less than 0.05. S. aureus distinguishes clearly between chlorhexidine and ordinary water as well as plain water and herbal. Cons show a significant difference between chlorhexidine and plain water. E. coli shows significant differences among all groups. Klebsiella shows a significant difference between (chlorhexidine-plain water). S. mutans shows a significant difference between chlorhexidine and plain water as well as plain water and herbal. There was no significant difference between natural mouthwash and chlorhexidine mouthwash (p = 0.786). However, there was a significant difference between chlorhexidine and water (p = 0.047) and between water and herbal mouthwash (p = 0.047).

On the inter-group comparison between baseline and the eighth post-operative day, PI and GI scores shows a statistically significant difference between all three groups p value, which was 0.000 (Table 6).

**Table 6:** Within-group comparison of GI score and PI score at baseline and on eighth postoperative day in patients using plain water, herbal, and chlorhexidine mouth washes * p value less than 0.05 is considered statistically significant. PI: Plaque index; GI: Gingival index

Plain water	Baseline Mean±SD	Eighth post-operative day Mean±SD	p value
PI score	1.9±0.11	1.3±0.18	0.000*
GI score	1.7±0.18	1.2±0.16	0.000*
Herbal			
PI score	1.7±0.34	0.8±0.30	0.000*
GI score	1.6±0.32	0.8±0.33	0.000*
Chlorhexidine			
PI score	1.9±0.30	0.5±0.10	0.000*
GI score	2.1±0.21	0.5±0.13	0.000*

## Discussion

Microorganisms of the oral microbiota can adhere to suture materials, a fact that favors their passage into the surgical wound and causes odontogenic infections and bacteremia. Suturing is very important in dental surgical procedures because effective wound closure is important for the success of any surgical procedure. A good suture goes a long way toward aesthetic as well as effective wound closure [3]. The accumulation of microorganisms on sutures may serve as a focus for odontogenic infections, which are caused by both aerobic and anaerobic bacteria, including species of staphylococcus, streptococcus, peptostreptococcus, prevotella, and bacteroids [4].

Effective antisepsis, appropriate antimicrobial prophylaxis, and wound contamination strategies must be implemented to reduce the risk of infection at the surgical site [3]. Post-operative care of a sutured wound is important after surgery. Sutured wounds in the oral cavity are kept clean through frequent rinses with either normal saline, chlorhexidine mouthwash, hydrogen peroxide diluted with saline, or fresh tap water [6].

The result of the present study demonstrated the adherence of a large number of microorganisms like S. aureus, coagulase-negative staphylococcus species (*Staphylococcus epidermis*), E. coli, Bacteroides, Klebsiella species, and S. mutans to the suture material after the periodontal surgery. In the present study, staphylococcus species were the most dominant species with the highest percentage of growth. In his 2003 study, Jussara Cia made similar observations.

In the present study, herbal and chlorhexidine were tested against the control group (plain water). Both chlorhexidine and herbal mouthwash showed a tendency for microbial reduction. With a mean of 2.28, the chlorhexidine group has the lowest microbial adherence to sutures, followed by the herbal group with a mean of 2.56 and the control group with a mean of 4.88. Previous research on the action of chlorhexidine on aerobic and anaerobic gram-positive and gram-negative bacteria found that chlorhexidine had a high affinity for microorganism cell walls and caused cell surface alterations that resulted in osmotic imbalance and cytoplasmic precipitation. Raquel et al. made similar experimental observations in 2009 [7]. Their study stated that both chlorhexidine and herbal mouthwash presented effective antimicrobial activity against the adherence of microorganisms to sutures, but herbal mouthwash was not as efficient as chlorhexidine mouthwash [7]. A statistically significant difference between the chlorhexidine mouthwash and the herbal group was observed.

In the present study, although the antimicrobial activity of chlorhexidine was more efficient than that of herbal mouthwash (HiOra^TM^), herbal mouthwash also showed a tendency to reduce the number of microorganisms. In the present study, the mean CFU of S. aureus between herbal and chlorhexidine mouthwash were found to be statistically non-significant (p-value= 0.786), while a statistically significant difference was found between chlorhexidine and plain water (p values of 0.047 and 0.016, respectively).

Chlorhexidine is, to date, the proven most effective anti-plaque agent. Its efficacy can be attributed to its bacteriostatic and bactericidal properties [16]. However, its prolonged use is limited due to local side effects, including extrinsic tooth and tongue brown staining, taste disturbances, enhanced supragingival calculus formation, and desquamation of the oral mucosa. On the other hand, herbal mouthwash, due to its natural ingredients, has not reported any side effects and can serve as a good alternative for patients who wish to avoid alcohol (e.g., xerostomia patients), sugar (e.g., diabetics), any artificial preservatives, and colors in their mouth rinses.

On the inter-group comparison between the baseline and the eighth post-operative day, PI and GI scores show a statistically significant difference between all three groups. Similar observations were made by Parwani et al. in 2013 [17]. In his experimental study, they stated that the lowest GI and PI scores were demonstrated by chlorhexidine, followed by herbal mouthwash, and the highest scores were achieved with normal saline. They concluded that 0.2% chlorhexidine gluconate remains the best anti-plaque agent. In the present study, a statistically significant difference was found in the PI and GI scores between the herbal and control (plain water) groups. Similar observations were also made by Vaish et al. and Scherer et al. in their studies, where they stated that herbal mouthwash had better efficacy when compared to normal distilled water [18,19].

The limitations of the study are that it is just related to microbial counts but clinical healing of the particular area should be considered for future studies. Only CFUs were evaluated specifically more amount of bacteria could be evaluated acts as a limitation. Also, more accurate polymerase chain reaction for bacterial estimation should be done.

## Conclusions

This research looked at the effectiveness of several types of mouthwash in preventing bacterial adhesion to sutures after periodontal surgery, including those containing chlorhexidine and those containing herbs. Bacterial colonies were found in all three groups. Both chlorhexidine and herbal mouthwashes are effective in reducing the microbial load over sutures and PI and GI scores compared to the control group (plain water). But herbal mouthwash was less effective when compared to chlorhexidine. Differences between our results and those of other studies may be related to better sample size and better culture techniques. Hence, further studies of these variables with better standardization are needed. Such studies will be very helpful in opening new vistas of research in this field.
